# Microbiome Analysis Reveals Microecological Balance in the Emerging Rice–Crayfish Integrated Breeding Mode

**DOI:** 10.3389/fmicb.2021.669570

**Published:** 2021-06-08

**Authors:** Yi Wang, Chen Wang, Yonglun Chen, Dongdong Zhang, Mingming Zhao, Hailan Li, Peng Guo

**Affiliations:** ^1^Institute of Agricultural Products Processing and Nuclear Agriculture Technology Research, Hubei Academy of Agricultural Sciences, Wuhan, China; ^2^College of Biology and Pharmacy, Three Gorges University, Yichang, China; ^3^Institute of Marine Biology, Ocean College, Zhejiang University, Zhoushan, China

**Keywords:** aquaculture environment, eco-agriculture, gut microbiota, microbial interaction, genetic network

## Abstract

The interaction between the microbial communities in aquatic animals and those in the ambient environment is important for both healthy aquatic animals and the ecological balance of aquatic environment. Crayfish (*Procambarus clarkii*), with their high commercial value, have become the highest-yield freshwater shrimp in China. The traditional cultivation in ponds (i.e., monoculture, MC) and emerging cultivation in rice co-culture fields (i.e., rice–crayfish co-culture, RC) are the two main breeding modes for crayfish, and the integrated RC is considered to be a successful rice-livestock integration practice in eco-agricultural systems. This study explored the ecological interactions between the microbial communities in crayfish intestine and the ambient environment, which have not been fully described to date. The bacterial communities in crayfish intestine, the surrounding water, and sediment in the two main crayfish breeding modes were analyzed with MiSeq sequencing and genetic networks. In total, 53 phyla and 1,206 genera were identified, among which Proteobacteria, Actinobacteria, Tenericutes, Firmicutes, Cyanobacteria, Chloroflexi, Bacteroidetes, Acidobacteria, RsaHF231, and Nitrospirae were the dominant phyla. The microbiota composition significantly differed between the water, sediment, and crayfish intestine, while it did not between the two breeding modes. We also generated a co-occurrence correlation network based on the high-confidence interactions with Spearman correlation ρ ≥ 0.75. In the genera co-correlation network, 95 nodes and 1,158 edges were identified, indicating significant genera interactions between crayfish intestine and the environment. Furthermore, the genera clustered into three modules, based on the different environments. Additionally, *Candidatus_Bacilloplasma, g_norank_f_Steroidobacteraceae, Dinghuibacter, Hydrogenophaga, Methyloparacoccus*, and *Defluviicoccus* had the highest betweenness centrality and might be important in the interaction between crayfish and the ambient environment. Overall, this study enhances our understanding of the characteristics of the microbiota in crayfish and their surrounding environment. Moreover, our findings provide insights into the microecological balance in crayfish eco-agricultural systems and theoretical reference for the development of such systems.

## Introduction

The crayfish (*Procambarus clarkii*) was originally found in the southeastern United States but was introduced into China in the late 1930s. Crayfishes dig burrows as refugia against environmental stresses such as dehydration, low temperature, lack of food, and predation, and thus exhibit strong environmental adaptability. Notably, damage to agricultural fields, including uprooting, plant fragmentation, seedling consumption, and interference with seed germination and seedling establishment, has made them a pest in many countries (Si et al., 2017). However, in recent years, as crayfish have become a table delicacy, their value has remarkably increased. In China, crayfish aquaculture has increased rapidly, and crayfish has become the highest-yield freshwater shrimp. From 2003 to 2018, the yield of crayfish increased more than 30 times. In 2018, crayfish aquaculture used an area of 1.12 million hectares, its yield was of 1.64 million tons, and its total economic output was of 369 billion yuan (Bureau of Fisheries et al., 2019).

There are two main breeding modes for crayfish, namely the traditional cultivation in pond (i.e., crayfish monoculture, MC) and emerging cultivation in rice co-culture fields (i.e., rice–crayfish co-culture, RC). In the latter, crayfishes are bred in waterlogged rice fields, and this mode takes advantage of the shallow water environment and the idle production period of rice paddies in winter (Si et al., 2017). Furthermore, the combination of agriculture and aquaculture raises the utilization and productivity rates of the fields. Thus, RC is favorable, considering the increasing shortage of farmland resources. In 2018, RC occupied 0.84 million hectares in China, accounting for 75.1% of the total crayfish aquiculture area (National Bureau of Statistics of China [NBSPRC], 2018). RC is one of the most popular rice–livestock integration practices (Yang et al., 2019). Nevertheless, due to the use of fertilizers in rice farming and the return of rice straw to the field in RCs, there is a concern about their influence on both the surrounding environment and crayfish health. For instance, it has been documented that organic compound accumulation negatively affects the ambient microbial equilibrium by enriching potential pathogens and reducing probiotics, thereby causing environmental stresses in aquaculture (Sun et al., 2020).

In contrast to terrestrial animals, aquatic animals are directly exposed to the ambient microbiota in aquaculture ecosystems. Environmental microbes not only directly influence aquatic animals but also influence their gut microbes. Recently, numerous studies found that environmental microbes, such as Proteobacteria (Hou et al., 2018), Chloroflexi (Björnsson et al., 2002; Lv et al., 2018), Acidobacteria (Dedysh and Damsté, 2018), Cyanobacteria (Zehr and Ward, 2002; Hou et al., 2018), and Nitrospirae (Zecchin et al., 2018), play important roles in matter and energy recycling, and some of them contribute to the decomposition of eutrophication pollution (Douterelo et al., 2004). Furthermore, many gut microbes are derived from the aquaculture environment, and the gut microbiota are strongly influenced by ambient microbial communities (Zhao et al., 2020). The internal microbiota plays crucial roles in the nutrition, immunity, and disease resistance of animals (Giatsis et al., 2015; Carbone and Faggio, 2016). A previous study reported that the ratio of Firmicutes to Bacteroidetes in shrimp intestines was positively related to lipid metabolism (Sun et al., 2020). Some Proteobacteria (Shin et al., 2015) and Tenericutes (Huang et al., 2020) were considered to be opportunistic pathogens of aquatic animals, while Actinobacteria might be a good candidate for screening native aquatic probiotics (Mahajan and Balachandran, 2012; Liberton et al., 2019). Therefore, a comprehensive comparison between the microbiota in the ambient environment and that in aquatic animals is critical for our understanding of the complex interactions between animals and their surroundings. In turn, the microbiota characteristics of aquatic animals and the ambient environment may serve as valuable indicators for the assessment of environmental conditions.

In freshwater aquaculture, the potential relationship between the microbial communities in crayfish intestine and those in the surroundings, and the differences between the microbial communities in the two main breeding modes, namely MC and RC, have seldom been studied. Therefore, in this study, we investigated the bacterial communities in water, sediment, and crayfish intestine samples obtained from these two breeding modes. In other words, this study explored the relationship between the microbial communities in crayfish intestine and those in the surrounding environment. In addition, the influence of RC on crayfish and their surroundings was evaluated by comparing the microbial communities in the RC and traditional MC breeding modes.

## Materials and Methods

### Study Area

The experiment was carried out in Jianli County (29.91°N, 112.63°E), Hubei province, China, located in the Middle–Lower Yangtze Valley Plain. This area is low and flat, densely covered with rivers and lakes, and is one of the main production regions for both rice and crayfish. The soil type is waterloggogenic paddy soil, which has developed from river and lake sediments. The average annual temperature is 16.3°C, with a frost-free period of 255 days. The average annual rainfall is 1,226 mm (Liu et al., 2010).

### Experimental Design and Plot Maintenance

Traditional crayfish breeding in the pond mode (MC) and rice–crayfish co-culture breeding in the rice paddy mode (RC) were investigated. To ensure consistency in the experimental conditions, the MC and RC were established five kilometers apart, in two stations on the same river. Three fields within the same station were selected for each breeding mode, and all the irrigation and aquaculture water was pumped from the river. Each subplot had a surface area of approximately 1,300 m^2^ and was surrounded with 0.3-m-high nylon nets to prevent the crayfish from escaping. The RC field included a center paddy and a surrounding crayfish gutter that was 3.0–4.0 m wide and 1.0–1.5 m deep.

In the MC, routine feeding management was performed. In the RC, the field was irrigated after the harvest of mid-season rice, and the crayfish returned to the paddy along with the irrigation water at the beginning of October. Meanwhile, an additional broodstock was added as appropriate. All the rice straw was left in the paddy without tilling, and the crayfish grew in the paddy until the second season. Mature crayfishes were harvested at the beginning of June, and immature crayfishes migrated to the gutter after drainage of the paddy. Mid-season rice was planted by mechanical rotary tilling and artificial transplantation. A shrimp feed supply of 500 kg was added from March to May of each year, and the rice plants were fertilized with 120.0 kg/hm^2^ N, 36.0 kg/hm^2^ P_2_O_5_, and 60.0 kg/hm^2^ K_2_O each season.

### Sample Collection

To evaluate the global effect of the emerging RC on farm ecology, we studied the end of the entire farming cycle. All crayfish, water, and sediment samples were collected from the six subplots when mature crayfishes were harvested at the beginning of June. In the RC, samples were taken from the crayfish gutter to mitigate disturbance of the shallow water environment in the paddy fields. Water samples (0.25 L) were taken from the surface, middle, and bottom of the aquaculture water, mixed together into one sample, and filtered through a 0.22-micrometer millipore filter (Millipore, Merck) for DNA extraction (Sun et al., 2020). Water samples from the MC were labeled as MCw1, MCw2, and MCw3, and those from the RC as RCw1, RCw2, and RCw3. Sediment samples (200 g) were collected with an S-shaped 5-point collection method within 5 cm from the surface mud (Fan et al., 2019). After mixing together into one sample, 50 g of the sediment sample was used for DNA extraction. Sediment samples obtained from the MC were labeled as MCs1, MCs2, and MCs3, and those from the RC as RCs1, RCs2, and RCs3. Twenty samples were taken from healthy crayfishes with similar weights (18.9 ± 2.6 g), and the scarfskin was sterilized with 75% ethanol (Huang et al., 2018). The intestines of these 20 crayfishes were then collected and pooled into one intestine sample for DNA extraction. Intestine samples from crayfish bred in the MC were labeled as MCc1, MCc2, and MCc3, and those from the fish bred in the RC as RCc1, RCc2, and RCc3. The final samples of water, sediment, and crayfish intestine were flash frozen with liquid nitrogen and stored at −80°C until DNA extraction.

### DNA Extraction, PCR Amplification, and MiSeq Sequencing

The total DNA of water, sediment, and crayfish intestine samples was extracted using the E.Z.N.A. Soil DNA kit (Omega, Norcross, GA) according to the manufacturer’s instructions. DNA quantity and purity were detected using a NanoDrop 2000 microspectrophotometer (Thermo Fisher, Waltham, MA), and DNA integrity was assessed using 1% agarose gels. The V3-V4 hypervariable region of 16S rRNA was amplified using the 338F (5′-ACT CCT ACG GGG AGG CAG CAG-3′) and 806R (5′-GGA CTA CHV GGG TWT CTA AT-3′) primers to investigate the bacterial communities (Han et al., 2020). High-throughput sequencing was performed in a paired-end model using the Illumina MiSeq PE300 platform (Majorbio, China).

### Bioinformatic and Statistical Analyses

Before proceeding with the analyses, raw data from the Illumina MiSeq platform were obtained with Flash (version 1.2.11)^1^ and demultiplexed and quality-filtered using Qiime (version 1.9.1)^2^. Operational taxonomic units (OTUs) were clustered with a 97% similarity threshold using the Uparse software (version 7.0.1090)^3^. To correct for uneven sequencing efforts, OTU abundance information was normalized according to the least sequence number (Li et al., 2018). The normalized OTU abundance data were then used to calculate the diversity and distance between samples. The taxonomy of each OTU was classified with the RDP Classifier (version 2.11)^4^, against the SILVA rRNA database (SSU132)^5^, using a confidence threshold of 70% (Amato et al., 2013; Quast et al., 2013).

Alpha-diversity indices were calculated to illustrate the complexity of each sample, based on the normalization OTUs and using Mothur (version v.1.30.2; Schloss et al., 2011)^6^, which included the community richness parameters Ace and Chao1 and diversity parameters, namely the Shannon and Simpson indices. Beta-diversity analyses, namely hierarchical clustering tree, heatmap, and principal coordinates analysis (PCoA) on OTUs levels, were performed based on the Bray-Curtis distance (Jiang et al., 2019). To compare the similarity of the microbial composition in the different samples, a dendrogram based on Bray–Curtis distances was generated using the unweighted pair-group method with arithmetic mean (UPGMA) and displayed at the top of the heatmap. Analysis of similarities (ANOSIM) and Adonis analysis were further performed to check the microbial community similarities between the MC and RC modes, and the significance levels between groups were determined with 999 permutations (Leung et al., 2016). Functional profiling was inferred from the 16S rRNA marker gene sequences using PICRUSt I (Blankenberg et al., 2010). That is, the presumptive functions of the microbial communities in the crayfish intestine and the surrounding environment, namely the water and sediment, were illustrated using PICRUSt. Moreover, the clusters of orthologous groups (COG) functions of the predicted genes were classified in the Eggnog databases.

Various network approaches were used to analyze the data sets. A bipartite co-occurrence network was generated using the treatments as source nodes and the OTUs as target nodes, with edges corresponding to the connection of particular OTUs with specific treatments, to visualize the associations between the genera and the different samples. We divided the 18 samples into six groups (MCs, RCs, MCw, RCw, MCc, and RCc), corresponding to three environments (water, sediment, and crayfish intestine). Species co-correlations were calculated based on Spearman’s rank correlation coefficient. The connections between two species that indicated a strong (i.e., Spearman correlation coefficient ρ ≥ 0.75) and significant (i.e., *P* < 0.05) correlation were reserved and a co-correlation network was generated in Gephi (version v.0.9.3).

All reported values were the average of triplicate results (i.e., mean ± SD). Differences between populations were analyzed using a one-way ANOVA, and results with P < 0.05 were considered significant. Statistical analyses were performed and visualized on the online platform I-Sanger (Majorbio, Shanghai, China)^7^, based on various R packages. The raw data of the 16S rDNA gene sequence reads from the 18 samples were deposited into the NCBI Sequence Read archive (SRA) database with the following BioProject accession number: PRJNA663764 (SRR12650255 to SRR12650272).

## Results

### Characteristics of 16S rRNA Sequencing and Microbial Community Diversity

The bacterial 16S rRNA genes of the 18 samples were sequenced to study the microbial communities of crayfish intestines and the cultured environment, water, and sediment. After quality filtering and assignment, a total of 715,020 high-quality sequences were obtained, and the read depth was between 32,991 and 44,928 reads per sample (39,723 ± 3,026; raw sequence data are presented in Supplementary Table 1). Then, uneven sequencing depths were normalized to 32,991 reads in all samples, which generated a total of 6,347 OTUs, ranging from 334 to 3,434.

**FIGURE 1:**
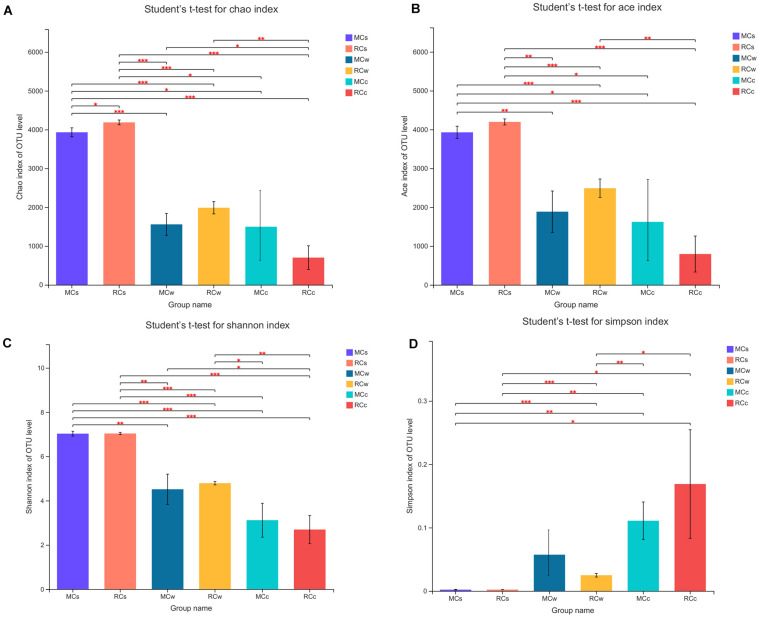
Differences in richness and diversity of bacterial species between sampling groups. **(A)** Chao1 index of OTU level. **(B)** Ace index of OTU level. **(C)** Shannon index of OTU level. **(D)** Simpson index of OTU level. Differences were assessed with Student’s *t*-test: * 0.01 < *P* ≤ 0.05; ** 0.001 < *P* ≤ 0.01; *** *P* ≤ 0.001.

The average Good’s coverage was 0.983342 (values ranged from 0.971 to 0.998) and indicated a saturated sequencing depth. The Ace and Chao1 indices were used to quantify species richness. The Ace index ranged from 371.882 to 4,277.969, and the Chao1 index ranged from 381.273 to 4,226.235 (Figure 1 and Supplementary Table 2). As for the Shannon and Simpson indices, they were used to calculate species diversity. The Shannon index ranged from 2.250 to 7.111, and the Simpson index ranged from 0.002 to 0.255 (Figure 1 and Supplementary Table 2). In general, the alpha diversity of the microbial community was similar between the two tested breeding modes but significantly differed according to the environment. The bacterial richness and diversity were the greatest in sediment, intermediate in water, and the lowest in crayfish intestine samples.

### Overall Microbial Communities in Water, Sediment, and Crayfish Intestine

**FIGURE 2:**
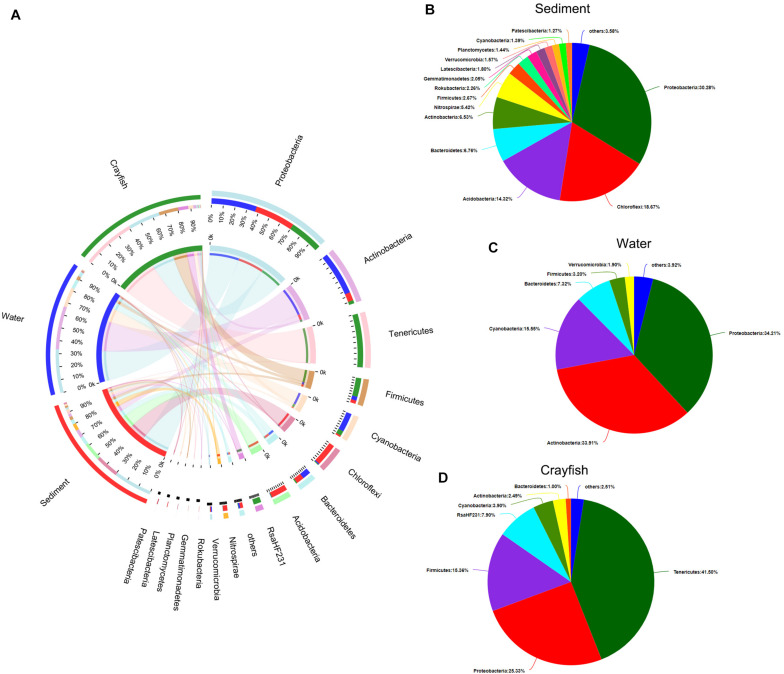
Phylum-level composition of the bacterial community in the sampling groups. **(A)** Distribution of phyla within the microbial community of each sample. The width of the bar of each phylum indicates the relative abundance of that phylum in the sample. **(B)** Composition of the bacterial community in sediment. **(C)** Composition of the bacterial community in water. **(D)** Composition of the bacterial community in crayfish intestine. Low abundance sequences (i.e., <1% abundance) were combined and specified as “others”.

A total of 53 phyla were identified in the bacterial microbial communities in crayfish intestine and the cultured environment, namely the water and sediment. Low-abundance sequences (i.e., with <1% abundance) were combined and specified as “others”. The top ten most abundant phyla, which accounted for approximately 93.4% of the total sequences, and their distribution across different categories (i.e., sediment, water, crayfish intestine – respective percentages are hereafter given in the same order) were as follows: Proteobacteria (33.71%; 38.09%; 28.20%), Actinobacteria (15.21%; 79.00%; 5.79%), Tenericutes (0.01%; 0.17%; 99.81%), Firmicutes (12.60%; 15.05%; 72.35%), Cyanobacteria (6.68%; 74.58%; 18.73%), Chloroflexi (92.71%; 3.98%; 3.31%), Bacteroidetes (44.81%; 48.54%; 6.64%), Acidobacteria (95.30%; 1.40%; 3.30%), RsaHF231 (0.00%; 0.13%; 99.87%), and Nitrospirae (95.73%; 1.38%; 2.89%; Figure 2A). The community pie charts in Figure 2 show the overall microbial communities at the phylum level in water (Figure 2B), sediment (Figure 2C), and crayfish intestine (Figure 2D). Proteobacteria was uniformly dispersed in the three habitats. Bacteroidetes was mainly dispersed in water and sediment. Sediment samples contained the highest abundance of Chloroflexi, Acidobacteria, and Nitrospirae, water samples contained the highest abundance of Actinobacteria and Cyanobacteria, and the crayfish intestine samples contained the highest abundance of Tenericutes, Firmicutes, and RsaHF231.

**FIGURE 3:**
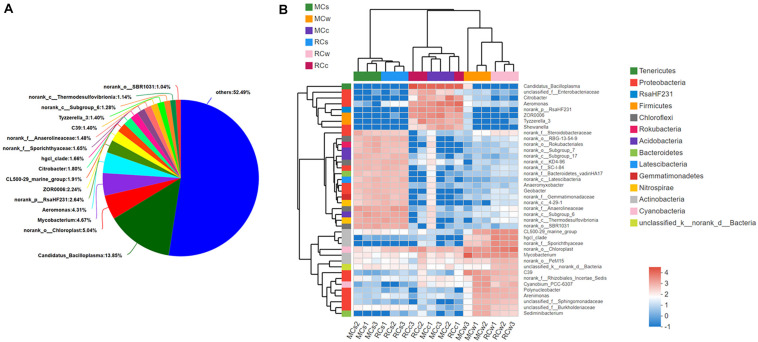
Genus-level composition of the bacterial community in the samples. **(A)** Overall bacterial community composition at the genus level (all samples pooled together). Low abundance sequences (i.e., <1% abundance) were combined and specified as “others.” **(B)** Distribution of the 40 most abundant genera across all samples. The log-transformed relative abundance of each genus in sample is depicted by color intensity. Bray–Curtis distances are shown at the top.

When the OTUs were analyzed at the genus level, a high diversity of microbes was identified. A total of 1,206 genera were detected across all 18 samples. Sixteen had a relative abundance greater than 1% and are shown in Figure 3A. Then, the top 40 most abundant genera were selected to generate a heatmap along with the clustering tree to visualize their distribution (Figure 3B). We observed that the samples from sediment, water, and crayfish intestine clearly clustered into three main branches in the sample dendrogram, which indicated a distinct difference in microbial community between the water, sediment, and crayfish intestine environments. The three main branches of the genera cluster tree also followed the sample categories, with the sediment branch, water branch, and crayfish intestine branch.

The characteristic dominant genera in the sediment were *norank_f__Steroidobacteraceae*, *norank_o__RBG-13-54-9*, *norank_o__Rokubacteriales*, *norank_o__Subgroup_7*, *norank_ c__Subgroup_17*, *norank_c__KD4-96*, *norank_f__SC-I-84*, *norank_f__Bacteroidetes_vadinHA17*, *norank_c__ Latescibacteria*, *Anaeromyxobacter*, *Geobacter*, *norank_f_ _Gemmatimonadaceae*, *norank_c__4-29-1*, *norank_f__ Anaerolineaceae*, *norank_c__Subgroup_6*, *norank_c__ Thermodesulfovibrionia*, and *norank_o__SBR1031*.

In the water, the characteristic dominant genera were *CL500-29_marine_group*, *hgcI_clade*, *norank_f__ Sporichthyaceae*, *norank_o__Chloroplast*, *Mycobacterium*, *norank_o__PeM15*, *unclassified_k__norank_d__Bacteria*, *C39*, *norank_f__Rhizobiales_Incertae_Sedis*, *Cyanobium_PCC-6307*, *Polynucleobacter*, *Arenimonas*, *unclassified_f__ Sphingomonadaceae*, *unclassified_f__ Burkholderiaceae*, and *Sediminibacterium*.

In crayfish intestine, the characteristic dominant genera were *Candidatus_Bacilloplasma*, *unclassified_f__Enterobacteriaceae*, *Citrobacter*, *Aeromonas*, *norank_p__RsaHF231*, *ZOR0006*, *Tyzzerella_3*, and *Shewanella*. Notably, microbial communities appeared to depend more on the sample category than on the breeding mode.

Furthermore, the Kruskal-Wallis H test was performed to check for significant differences in the microbes among habitats at both phylum and genus levels, and the results are shown in Supplementary Figure 2.

### ANOSIM/Adonis Analysis of the Differences in Microbial Communities Between Two Breeding Modes

ANOSIM and Adonis analysis revealed significant differences between the clusters of water, sediment, and crayfish intestine (Table 1 and Supplementary Figure 1) at the OTU level. In contrast, ANOSIM and Adonis analysis suggested that the difference between the MC and RC modes in all water, sediment, and crayfish intestine samples at the OTU level was not statistically significant (Table 1 and Supplementary Figure 1). These results indicate that the RC breeding mode had no significant influence on the microbial communities of both crayfish intestines and their cultural surroundings compared with the traditional MC breeding mode.

**TABLE 1:** ANOSIM/Adonis analysis.

Group	ANOSIM^*a*^			Adonis^*b*^		
	R^*c*^	P	Permutation_num	F. model	R^2 d^	Pr (> F)^*e*^
Sediment vs. Water vs. Crayfish-intestine	1	0.001	999	19.9878	0.7272	0.001
MC-sediment vs. RC-sediment	0.5556	0.098	999	3.1507	0.4406	0.1
MC-water vs. RC-water	0.5556	0.098	999	5.5567	0.5814	0.1
MC-crayfish-intestine vs. RC-crayfish-intestine	0.3333	0.199	999	1.6891	0.2969	0.2

*^*a*^Analysis of similarities (ANOSIM) was performed based on Bray-Curtis distances. ^*b*^Adonis is a non-parametric statistical method. ^*c*^An R value closer to 1 indicates a greater difference between groups. ^*d*^The R^2^ value shows the percentage of variation explained by the supplied mapping file category. ^*e*^Pr (> F) is the p-value representing statistical significance. Permuation_num: number of permutations; MC: monoculture mode; RC: rice-crayfish co-culture mode.*

### Relationship Between Microbial Communities in Water, Sediment, and Crayfish Intestines

**FIGURE 4:**
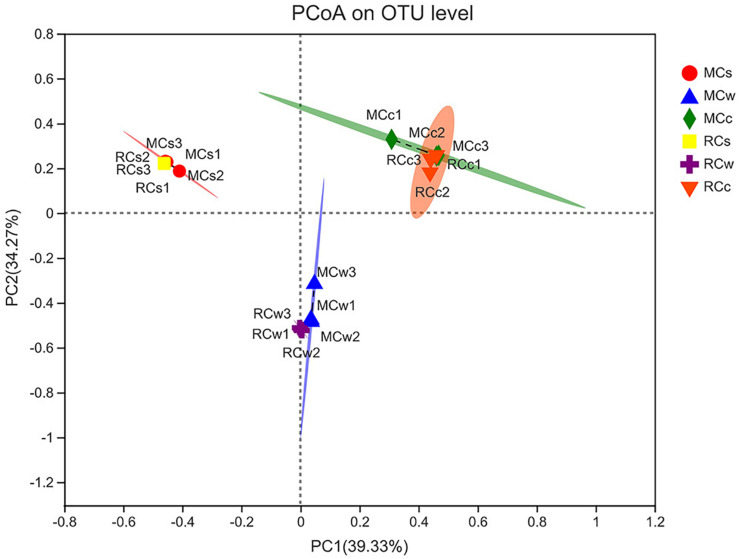
Principal co-ordinates analysis (PCoA) of the bacterial community at the level of operational taxonomic units (OTUs).

The microbial communities of each sample clustered with PC1 = 39.33% and PC2 = 34.27% of the total variation (Figure 4). Moreover, samples of the same category were clustered together, which was consistent with the UPGMA tree. Additionally, although samples from the different breeding modes were separated to some extent, their 95% confidence ellipses crossed in all water, sediment, and crayfish intestine samples.

**FIGURE 5:**
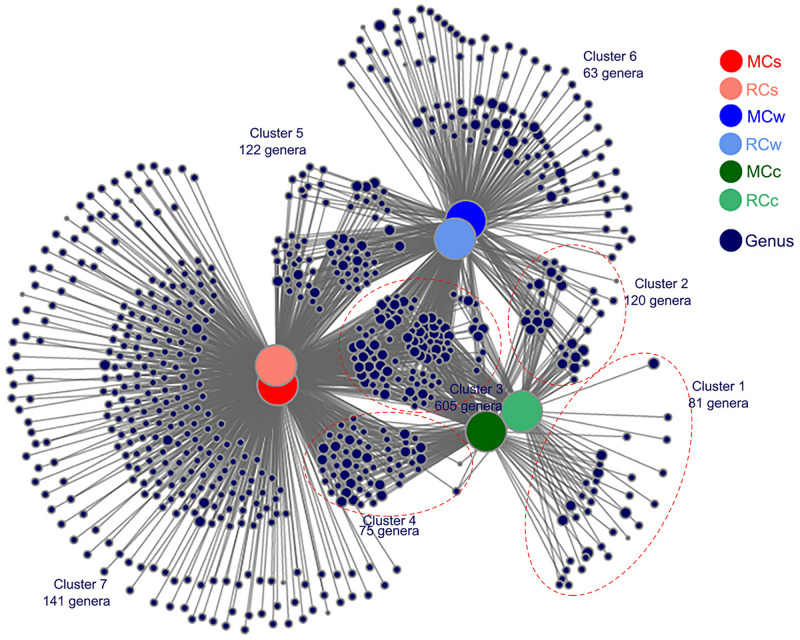
Bipartite co-occurrence network showing the associations between genera and the different samples. Genera with ≥5 sequence numbers were reserved. Node sizes represent the relative abundance of the genera in the data sets, with bigger nodes indicating greater abundances. Edges represent the association patterns of individual genus with the sampling groups.

A total of 1,207 nodes were identified in the bipartite co-occurrence network; 23.61% (285 genera, clusters 1, 6, and 7) of the sampled genera were most strongly associated with only one environment (Figure 5), confirming the basic distinctness of the microbial communities in various systems. Furthermore, 26.26% (317 genera, clusters 2, 4, and 5) of these genera were associated with two environments, and 50.12% (605 genera, cluster 3) were associated with all three environments (Figure 5), indicating a strong correlation among microbial communities in water, sediment, and crayfish intestines. In particular, the distribution of crayfish intestine microbes (a total of 881 genera) suggested their strong correlation with the aquatic environment. Only 9.19% (81 genera, cluster 1) of crayfish intestine microbiota were unique to crayfish intestines, 82.29% (725 genera, clusters 2 and 3) were shared between crayfish intestines and water, 77.19% (680 genera, clusters 3 and 4) were shared between crayfish intestines and sediment, and 68.67% (605 genera, cluster 3) were shared among the three habitats (Figure 5). In addition, the bipartite network also showed that samples from the same environment shared a large number of genera (Figure 5), suggesting that the breeding mode had little influence on the microbial communities.

**FIGURE 6:**
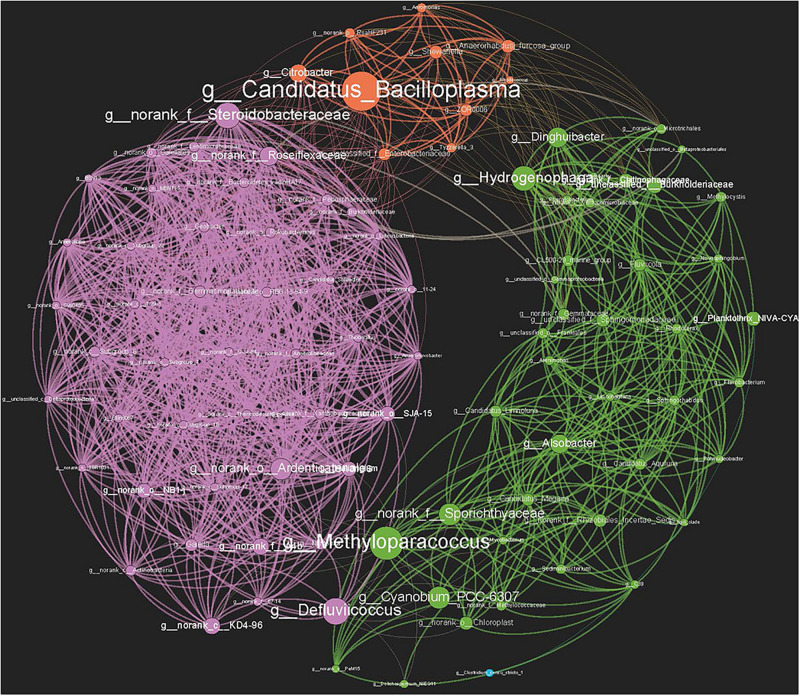
Co-correlation network of bacterial genera based on Spearman correlation. High-confidence interactions with Spearman correlation ρ ≥ 0.75 were reserved. Three main modules, which correspond to the dominant genera in sediment, water, and crayfish intestine, respectively, were generated with a 0.409 modularity index. All networks are displayed as nodes (genera) and edges (significant interactions among genera nodes). The same node colors represent genera belonging to the same module. Purple: sediment module; green: water module: orange: crayfish intestine module. Node sizes indicate the betweenness centrality of the genera in the data sets.

A total of 95 nodes and 1,158 interactions were identified in the genera co-correlation network (Figure 6), and the average clustering coefficient was 0.712. The transitivity of the network was 0.777, the network diameter was 6, and the average path length was 2.279. A high level of connectivity indicated significant interactions between the genera in crayfish intestines and the environment. Furthermore, the genera from different environments clustered into three modules in the network, based on their characteristic dominant genera (Figures 3B, 6). The modularity index was 0.409, indicating a valid modular structure in the network (Newman, 2006). Notably, the genera present in the sediment had the tightest cluster, while those found in crayfish intestine displayed frequent connections with the genera encountered in water. To identify the potential key genera connecting different environments, we focused on the hubs with the highest betweenness centrality parameters (Lupatini et al., 2014). *Candidatus_Bacilloplasma* (degree 29, clustering 0.468), which belongs to the phylum Tenericutes, had the highest betweenness centrality (i.e., 446.2839). As a genus mainly distributed in crayfish intestine, the presence of *Candidatus_Bacilloplasma* was strongly correlated with both water and sediment clusters. The genus *g_norank_f_Steroidobacteraceae* (degree 43, clustering 0.658), phylum Proteobacteria, was mainly distributed in sediment that exhibited a strong correlation with the crayfish intestine cluster, which had a 253.365 betweenness centrality. The genera *Dinghuibacter* (degree 23, clustering 0.459), belonging to the phylum Bacteroidetes, and *Hydrogenophaga* (degree 25, clustering 0.467), phylum Proteobacteria, were distributed in both water and crayfish intestines and might be the key nodes connecting the water and crayfish intestine clusters, which had 171.590 and 240.221 betweenness centrality, respectively. As for the genera *Methyloparacoccus* (degree 20, clustering 0.421) and *Defluviicoccus* (degree 19, clustering 0.409), both belonging to the phylum Proteobacteria, they were distributed in both water and sediment and might be the key nodes connecting the water and sediment clusters, which had 359.762 and 265.153 betweenness centrality, respectively (Figure 6 and Supplementary Table 3).

### PICRUst Functional Prediction of Microbial Communities

PICRUSt is a computational approach to predict the functional composition of a metagenome using marker gene data and a database of reference genomes (Langille et al., 2013). The dominant functions of the microbial communities in the crayfish intestine and the surrounding environment were as follows: energy production and conversion (relative abundance: 6.60–7.60%); amino acid transport and metabolism (7.84–8.25%); nucleotide transport and metabolism (2.34–2.86%); carbohydrate transport and metabolism (5.60–6.12%); coenzyme transport and metabolism (4.07–4.23%); lipid transport and metabolism (3.48–6.06%); translation, ribosomal structure, and biogenesis (5.11–6.68%); transcription (5.38–7.47%); replication, recombination, and repair (5.35–6.73%); cell wall, membrane, and envelope biogenesis (6.11–7.46%); inorganic ion transport and metabolism (5.35–6.13%); secondary metabolite biosynthesis, transport, and catabolism (1.78–3.66%); general function prediction only (8.37–8.70%); signal transduction mechanisms (5.65–7.82%); and post-translational modification, protein turnover, and chaperones (3.97–4.44%).

**FIGURE 7:**
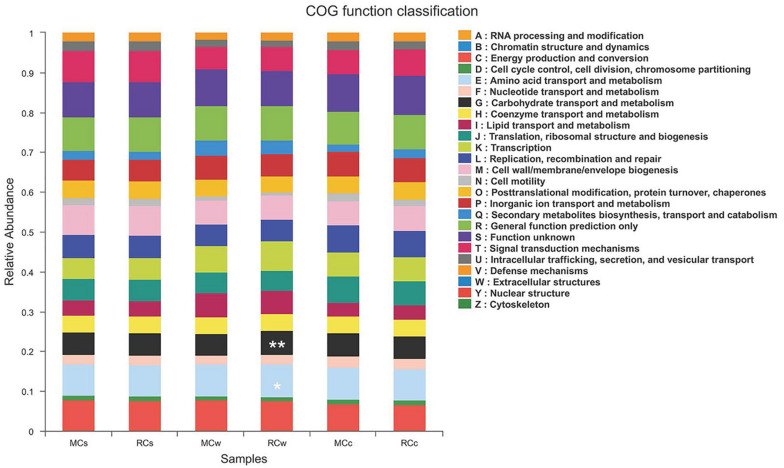
Clusters of orthologous group (COG) function classification of the six sampling groups. * indicate COG functions significant difference between MCw and RCw. * 0.01 < *P* ≤ 0.05; ** 0.001 < *P* ≤ 0.01.

Notably, the RC mode had a higher abundance, compared with that in the MC mode, of functions related to the transport and metabolism of carbohydrates (6.12 vs. 5.38%), amino acids (8.25 vs. 8.07%), and nucleotides (2.37 vs. 2.34%) in water samples (Figure 7). In contrast, the RC mode had a lower abundance in functions related to lipid transport and metabolism in water samples than did the MC mode (3.48 vs. 3.79%). These results imply differences in metabolism between the two modes.

## Discussion

Food security is becoming a global problem with the rapid increase in the world population. Eco-agricultural systems are considered to be a good strategy to face the challenges of land limitation, water scarcity, and climate change. As one of the most popular rice-livestock integration practices, the rice–crayfish co-culturing system has been developed rapidly in recent years (Yang et al., 2019). Previous studies have shown that eco-agricultural systems contribute to increasing the use of ecological processes and biodiversity, and reduce the rate of chemical fertilizer and pesticide application (Zhang et al., 2015). However, there is concern regarding the influence of the returning rice straw on the surrounding environment because organic compound accumulation might negatively affect the ambient microbial equilibrium by enriching potential pathogens and reducing probiotics, thereby causing environmental stress in aquaculture systems (Sun et al., 2020). In this work, we attempted to apply the microbial community as an indicator for calculating the ecological balance.

### Overview of the Dominant Bacterial Taxa

The dominant phyla in our study included Proteobacteria, Actinobacteria, Tenericutes, Firmicutes, Cyanobacteria, Chloroflexi, Bacteroidetes, and Acidobacteria, and their relative abundances varied between crayfish intestine and the surrounding water and sediment, which is consistent with the results of previous studies on shrimp and other aquatic livestock (Hou et al., 2018; Fan et al., 2019). The crucial roles of these phyla in nutrient cycling, water quality control, and the fitness of aquaculture animals have been well established, and thus, discussion on this aspect is not repeated here.

RsaHF231 and Nitrospirae were within the top ten dominant phyla, but as yet, their role in aquaculture had seldom been studied. Nitrospirae had a significantly higher abundance in sediment samples than in water and crayfish intestines. Nitrospirae have been detected in rice paddies, and they are known to have a versatile metabolism, which includes processed such as the chemolithoautotrophic use of ammonia and nitrite, hydrogen oxidation coupled to oxygen respiration, and formate-driven nitrate respiration to nitrite (Zecchin et al., 2018). RsaHF231 was the most abundant in crayfish intestine. The relative abundance of RsaHF231 in crayfish intestine was of 7.90%, whereas it was of 0.01% in water. RsaHF231 was found in the guts of black soldier fly in a previous study (Jiang et al., 2019), and we speculate that the presence of RsaHF231 in the former might have originated from the black soldier fly larvae meal fed to the crayfish.

### Microbial Interactions Between Crayfish and the Surroundings

Co-correlation networks are considered to be a powerful tool for exploring taxa coexistence in complex microbial communities (Jiang et al., 2019). The study of the topological properties of the co-correlation network provided direct and valuable information on the microbial interaction between crayfish and their surroundings. Furthermore, modularity has been suggested to be an important attribute of ecosystem stability and community resilience (Olesen et al., 2007), and modules in a co-correlation network can potentially indicate direct or indirect interactions between microbes. These interactions may also be due to shared niches or a high level of phylogenetic relatedness (Faust and Raes, 2012). It is worth noting that many bacterial taxa with low abundance could also play disproportionately large roles in niches and functions (Sun et al., 2018). Based on these theories, we could find the key microbes that connect various environments.

In this study, *Candidatus_Bacilloplasma* (phylum Tenericutes) was the most central genus in the co-correlation network. Tenericutes is a phylum of bacteria that lack a peptidoglycan cell wall, and the most well-studied clade in this phylum is the class Mollicutes (Wang et al., 2020). Notably, Tenericutes have been detected in the gut of various aquatic organisms, and some environmental Tenericutes are considered to be pathogens (Huang et al., 2020). Nevertheless, previous research has also shown that Tenericutes can be mutualistic symbionts in the gut of their host species (Wang et al., 2020). For instance, Tenericutes play a role in the degradation of recalcitrant C sources in the stomach and pancreas of isopods (Wang et al., 2004). However, their genomes are linked to an extreme reduction in metabolic capacity resulting from a lack of genes that are related to regulatory elements, biosynthesis of amino acids, and intermediate metabolic compounds, which must be imported from the host. Tenericutes might serve as an important indicator of host homeostasis and health (Wang et al., 2020). A previous study on *Litopenaeus vannamei* reported that *Candidatus_Bacilloplasma* and *Psychromonas* are the only two dominant genera in the shrimp intestine and are correlated with physiological parameters in shrimp (Huang et al., 2018). In addition, a recent study has also linked *Candidatus_Bacilloplasma* enrichment and the disorder of metabolic functions with white feces syndrome in shrimp (Wang et al., 2020). It is obvious that as a mutualistic symbiont, *Candidatus_Bacilloplasma* plays an important role in the microbial interaction of crayfish with the ambient environment, which mainly consists of water and sediment.

Environmental bacteria also strongly connect with the internal microbiota of crayfish. In our study, most of the central hubs belonged to the phylum Proteobacteria. Proteobacteria was the most abundant phylum and was uniformly dispersed in the three environments (sediment, water, and crayfish intestines). The co-correlation network indicated that Proteobacteria, such as those from the genera *Methyloparacoccus*, *Defluviicoccus*, *Hydrogenophaga*, and *g_norank_f_Steroidobacteraceae*, played an important role in the interaction between crayfish and the environment. In addition, the genus *Dinghuibacter*, which belongs to the phylum Bacteroidetes, also corresponded to an important node that connected the water and crayfish intestine samples. As one of the most abundant phyla in aquatic bacteria, Proteobacteria can inhabit various environments and play crucial roles in matter and energy recycling. However, as an abundant generalist colonizer, the abnormal increase in Proteobacteria often generates dysbiosis of the microbial composition in the host and leads to disease (Shin et al., 2015). Thus, Proteobacteria might serve as an important marker of healthy aquaculture.

Furthermore, the PICRUst functional prediction might have provided a microbiological explanation for the microbial connection between crayfish and their surroundings. Functions related to carbohydrate, amino acid, lipid, and inorganic ion transport and metabolism, as well as energy production and conversion, DNA replication and reparation, among others, were found in crayfish and the ambient microbiota. We speculate that diversity functions play an important role in the metabolism of the extra materials brought by the integrated RC breeding mode and maintain a stable microbiota structure in RCs, thus resulting in inconspicuous differences in the microbiota composition between the MC and RC breeding modes. Accordingly, the two testing modes also had little differences in environmental physico-chemical properties. The pH, dissolved oxygen, and ammonia nitrogen were similar between MC and RC modes; nitrate nitrogen and COD were slightly higher in RC mode than MC mode (Supplementary Table 4). Therefore, the functional diversity of material and energy metabolism guarantees the microecological balance in crayfish eco-agricultural systems.

Although the difference between the microbial structure in the MC and RC was not statistically significant, we still found traces of the influence of the rice–crayfish co-culturing system on the original crayfish ecosystem from PICRUst functional prediction. This might have been caused by the additional planting of rice and the return of rice straw in the RC mode compared with the MC mode. There was a higher abundance of functions related to the transport and metabolism of carbohydrates, amino acids, and nucleotides in the water samples from the RC than in those from the MC. This may be explained by the fact that an increase in these functions facilitates the degradation of rice straw. In contrast, functions related to lipid transport and metabolism were less abundant in the water samples from the RC than in those from the MC. We speculate that this might be caused by the higher amount of formulated diet, which has a high fat content, that is fed to crayfish in MC.

Overall, the microbial communities of the RC mode had no significant differences with those of the traditional MC mode. Decomposer microbes play a crucial role in maintaining ecological balance in eco-agricultural systems. The shift in PICRUst predicted functions reflected the action of the microbes upon crop materials brought by the plants in the RC mode. The co-correlation networks showed the diversity and complex interactions of microbial communities among crayfishes and the aquacultural environment, which may guarantee the robustness of the ecological system and also cause the similar microbial structures observed between the MC and RC modes. Evidently, the microbiome constitutes a promising indicator for calculating ecological balance. In particular, the hub microbes in the interaction network between crayfishes and the ambient environment, such as *Candidatus_Bacilloplasma*, Proteobacteria, and Bacteroidetes, are of particular concern in the micro-ecological environment of crayfish farming.

## Conclusion

The present study analyzed the profiles of microbial communities and highlighted the characteristic bacterial composition in crayfish, water, and sediment, respectively. Furthermore, we established the relationship between the bacterial communities in crayfish intestine and the ambient environment and provided microbial evidence of their interaction. Moreover, this study showed that the rice–crayfish co-culture had little influence on the microecological environment compared with the traditional crayfish monoculture, and provided insight into the microecological balance in crayfish eco-agricultural systems even with the additional ecological load. That is, the additional planting of rice and return of rice straw in the RC mode did not disturb the microbial interactions between crayfishes and the ambient environment. However, it should be noted that this study observed only a snapshot at the end of the entire farming cycle and a limited number of samples, which were limitations of our study. Further studies are still needed on the interaction between rice stalks and shrimp as well as the impact on water quality and the environment during the decomposition process of the returning rice stalk residue. Overall, this study provided a theoretical reference to direct crayfish farming and the development of crayfish eco-agricultural systems.

## Data Availability Statement

The raw data of the 16S rDNA gene sequence reads from the 18 samples were deposited into the NCBI Sequence Read archive (SRA) database with the following BioProject accession number: PRJNA663764 (SRR12650255 to SRR12650272).

## Author Contributions

YW: methodology, conceptualization, writing – original draft, raft, and writing – review. CW: investigation and formal analysis. YC: conceptualization and investigation. DZ and HL: soft-ware and data curation. MZ: visualization and data curation. PG: supervision and project administration. All authors contributed to the article and approved the submitted version.

## Conflict of Interest

The authors declare that the research was conducted in the absence of any commercial or financial relationships that could be construed as a potential conflict of interest.

## Footnotes

1 https://ccb.jhu.edu/software/FLASH/index.shtml

2 http://qiime.org/install/index.html

3 http://drive5.com/uparse

4 https://sourceforge.net/projects/rdp-classifier/

5 https://www.arb-silva.de/

6 https://www.mothur.org/wiki/Download_mothur

7 https://www.i-sanger.com/

## Supplementary Material

Supplementary Figure 1 ANOSIM analysis of the samples.

Supplementary Figure 2 Kruskal-Wallis H test bar plot among habitats on both phylum and genus level.

Supplementary Table 1 OUT reads number raw data of all samples.

Supplementary Table 2 Alpha-diversity indices of all samples.

Supplementary Table 3 Attributes of the co-correlation network.

Supplementary Table 4 The environmental physico-chemical properties in MC and RC breeding modes.
