# Supplementary figures and images for: Microbiome Analysis Reveals Microecological Balance in the Emerging Rice–Crayfish Integrated Breeding Mode
Source: Front Microbiol. 2021 Jun 8;12:669570. doi: 10.3389/fmicb.2021.669570 (PMC8219076; doi:10.3389/fmicb.2021.669570)

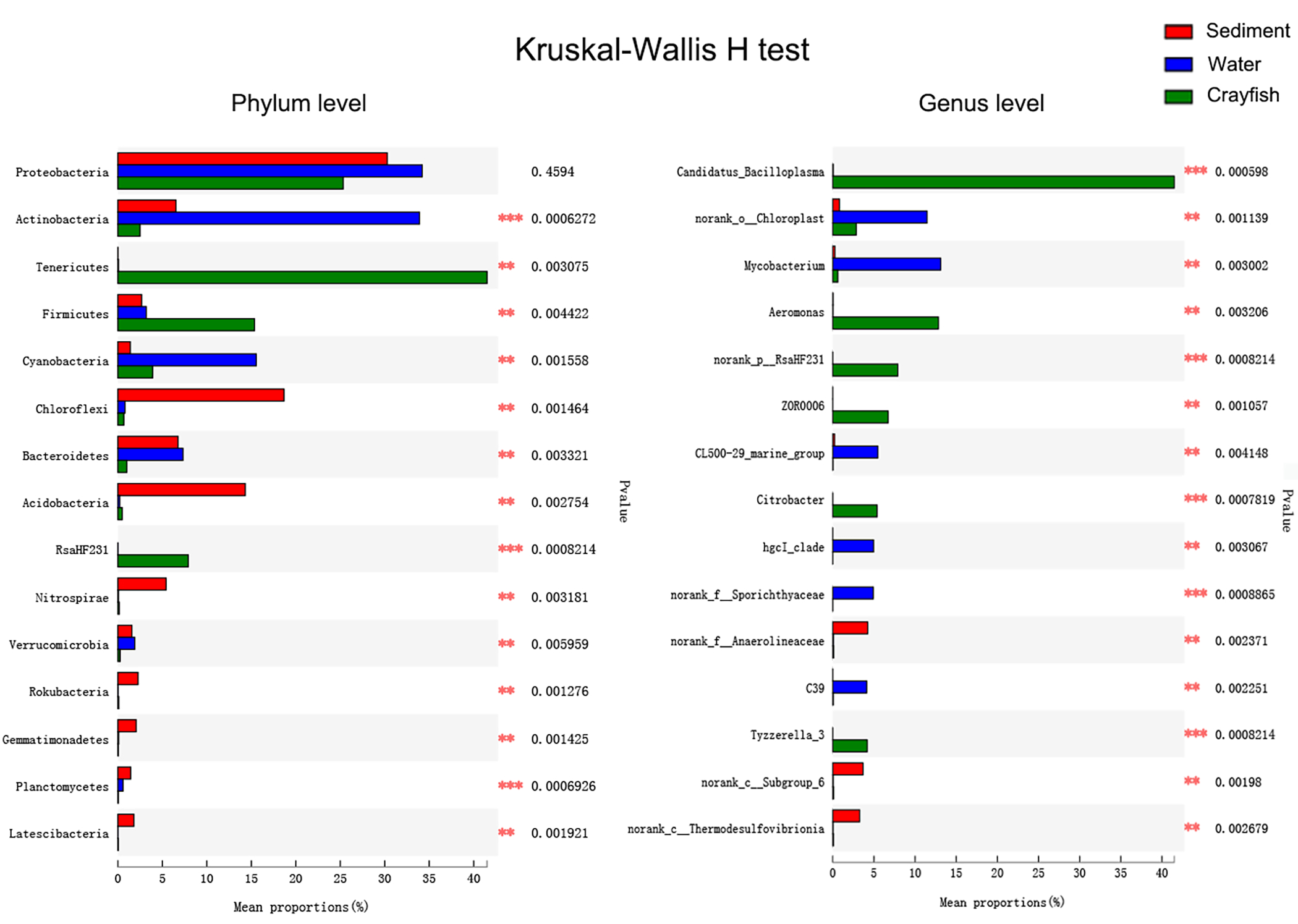

Supplement: Supplementary Figure 1 — ANOSIM analysis of the samples. [file Image_1.JPEG]
