# Supplementary material for: Microbiome Analysis Reveals Microecological Balance in the Emerging Rice–Crayfish Integrated Breeding Mode
Source: Front Microbiol. 2021 Jun 8;12:669570. doi: 10.3389/fmicb.2021.669570 (PMC8219076; doi:10.3389/fmicb.2021.669570)
Supplement: Supplementary Figure 2 — Kruskal-Wallis H test bar plot among habitats on both phylum and genus level. [file Data_Sheet_1.DOCX]

Figure S1 ANOSIM analysis of the samples. A. ANOSIM analysis between the three environment, i.e. sediment vs. water vs. crayfish intestine; B. ANOSIM analysis between sediment samples for MC (MCs) and RC (RCs); C. ANOSIM analysis between water samples for MC (MCw) and RC (RCw); D. ANOSIM analysis between crayfish intestine samples for MC (MCc) and RC (RCc)
