# Supplementary material for: Microbiome Analysis Reveals Microecological Balance in the Emerging Rice–Crayfish Integrated Breeding Mode
Source: Front Microbiol. 2021 Jun 8;12:669570. doi: 10.3389/fmicb.2021.669570 (PMC8219076; doi:10.3389/fmicb.2021.669570)
Supplement: Supplementary Table 4 — The environmental physico-chemical properties in MC and RC breeding modes. [file Table_4.DOCX]

Table S4. The environmental physico-chemical properties in MC and RC breeding modes

|  | MC | RC |
| --- | --- | --- |
| pH | 7.32 ± 0.14 a* | 7.36 ± 0.26 a |
| Dissolved oxygen (mg/L) | 7.73 ± 0.43 a | 7.67 ± 0.29 a |
| NH_4_^+^ -N (mg/L) | 0.49 ± 0.13 a | 0.48 ± 0.09 a |
| NO_3_^+^ -N (mg/L) | 0.32 ± 0.04 a | 0.35 ± 0.06 b |
| TP (mg/L) | 0.42 ± 0.11 a | 0.44 ± 0.39 a |
| COD (mg/L) | 41.35 ± 5.26 A | 46.36 ± 5.72 B |

* The data represent the mean and standard deviation from triplicate independent experiments. Values not sharing common letters are significantly different (one-way ANOVA with the Student-Newman-Keuls method, P < 0.05). The lowercase indicates 0.01< P ≤ 0.05, and uppercase indicates P ≤ 0.01.
